# *CACNA1D* De Novo Mutations in Autism Spectrum Disorders Activate Cav1.3 L-Type Calcium Channels

**DOI:** 10.1016/j.biopsych.2014.11.020

**Published:** 2015-05-01

**Authors:** Alexandra Pinggera, Andreas Lieb, Bruno Benedetti, Michaela Lampert, Stefania Monteleone, Klaus R. Liedl, Petronel Tuluc, Jörg Striessnig

**Affiliations:** a Department of Pharmacology and Toxicology, Center for Molecular Biosciences, University of Innsbruck, Innsbruck, Austria.; b Institute of General, Inorganic and Theoretical Chemistry, Center for Molecular Biosciences, University of Innsbruck, Innsbruck, Austria.

**Keywords:** Autism spectrum disorders, Calcium channel blockers, Human genetics, L-type calcium channels, Neuropsychiatric disorders, Whole-exome sequencing

## Abstract

**Background:**

Cav1.3 voltage-gated L-type calcium channels (LTCCs) are part of postsynaptic neuronal signaling networks. They play a key role in brain function, including fear memory and emotional and drug-taking behaviors. A whole-exome sequencing study identified a de novo mutation, p.A749G, in Cav1.3 α_1_-subunits (*CACNA1D*), the second main LTCC in the brain, as 1 of 62 high risk–conferring mutations in a cohort of patients with autism and intellectual disability. We screened all published genetic information available from whole-exome sequencing studies and identified a second de novo *CACNA1D* mutation, p.G407R. Both mutations are present only in the probands and not in their unaffected parents or siblings.

**Methods:**

We functionally expressed both mutations in tsA-201 cells to study their functional consequences using whole-cell patch-clamp.

**Results:**

The mutations p.A749G and p.G407R caused dramatic changes in channel gating by shifting (~15 mV) the voltage dependence for steady-state activation and inactivation to more negative voltages (p.A749G) or by pronounced slowing of current inactivation during depolarizing stimuli (p.G407R). In both cases, these changes are compatible with a gain-of-function phenotype.

**Conclusions:**

Our data, together with the discovery that Cav1.3 gain-of-function causes primary aldosteronism with seizures, neurologic abnormalities, and intellectual disability, suggest that Cav1.3 gain-of-function mutations confer a major part of the risk for autism in the two probands and may even cause the disease. Our findings have immediate clinical relevance because blockers of LTCCs are available for therapeutic attempts in affected individuals. Patients should also be explored for other symptoms likely resulting from Cav1.3 hyperactivity, in particular, primary aldosteronism.

L-type calcium channels (LTCCs; Cav1) are one of the three major classes (Cav1–Cav3) of voltage-gated calcium channels (1). They are expressed in most electrically excitable cells (1–3). Many body functions, including muscle contraction and brain, endocrine, and sensory functions, depend on proper LTCC activity (2–4). The LTCCs contain high-affinity drug-binding sites for different chemical classes of organic calcium channel blockers (5). Blocking of LTCCs in vascular smooth muscle and the heart has been therapeutically used for decades to treat elevated blood pressure and cardiac ischemias. The dihydropyridine class of LTCC blockers still belongs to the top-selling antihypertensives.

Despite their importance as peripheral drug targets, LTCCs play a key role for normal brain function. Within the LTCC family (Cav1.1–Cav1.4), Cav1.2 and Cav1.3 are the two isoforms expressed in the brain (3). They are located at postsynaptic somatodendritic sites, shape short-term and long-term adaptations of synaptic function (2,4,6,7), and are often present in the same neurons (6). However, despite high structural homology, they differ with respect to their gating properties and protein interaction partners (3). They contribute differently to various brain functions, such as emotional and drug-taking behaviors and different types of memory (2–4). Cav1.3 comprises only ~10% of the LTCCs in the brain (8), but because of its more negative activation voltage range, it can carry inward calcium currents at threshold voltages (9,10), shaping neuronal firing patterns and contributing to pacemaker currents (2,6,7,11).

Data from mouse studies and human channelopathies provide important insight into the potential role of Cav1.2 and Cav1.3 LTCCs in human brain disease. In genome-wide association studies and exome sequencing studies, *CACNA1C* has emerged as a new candidate gene for neuropsychiatric disease, including bipolar disorder, major depression, schizophrenia, and autism (12–15). Reduced Cav1.2 expression in the mouse forebrain results in anxiety-like behavior. Decreased channel function may contribute to the pathophysiology of anxiety in neuropsychiatric diseases (16). Timothy syndrome is a rare multiorgan disorder resulting from Cav1.2 gain-of-function mutations (OMIM No. 601005) (17), and surviving patients may also develop autism and epilepsy (17). Knock-in mice expressing the human mutation replicate autistic behavioral traits (18). Both gain and loss of Cav1.2 channel activity can lead to central nervous system dysfunction.

In contrast, heterozygous loss of Cav1.3 channel function does not result in a detectable phenotype in mice (3,19) and humans (20). Instead, the specific acute activation of Cav1.3 induces depression-like behaviors (8) and leads to activation of brain regions involved in anxiety and fear circuits (21). Gain-of-function of this channel may also underlie neuropsychiatric symptoms in humans. This possibility is further supported by the description of two patients with two different germline *CACNA1D* gain-of-function mutations (22,23). These mutations caused a severe congenital multiorgan syndrome with primary aldosteronism (22,23), seizures, and neurologic abnormalities (PASNA; OMIM No. 615474). Symptoms also included global developmental delay and intellectual disability (23) indicating that constitutively enhanced Cav1.3 activity interferes with normal neuronal function and development (22,23).

In the present study, we show that one *CACNA1D* mutation (p.A749G in Cav1.3 α1), which has been reported as 1 of 62 high risk–conferring mutations in a whole-exome sequencing (WES) study of patients with sporadic autism and intellectual disability (24), induces a strong increase in Cav1.3 channel function. We screened WES data in patients with sporadic autism for other *CACNA1D* de novo mutations and identified p.G407R in another patient, for which we also demonstrate a pronounced gain-of-function. Our data strongly support *CACNA1D* as a recurrent risk gene for autism spectrum disorder (ASD). Given the nature of the mutation, the severe congenital disorder in two other patients, and the pathophysiologic relationship to Timothy syndrome, our data strongly suggest possibly a causal role of *CACNA1D* gain-of-function mutations for ASD in these patients. This observation has immediate clinical relevance because clinically used blockers of LTCCs are available for immediate therapeutic intervention. Affected patients should also be monitored for other symptoms expected from Cav1.3 hyperactivity, in particular, primary hyperaldosteronism and hypertension (22,23).

## Methods and Materials

### WES Data Analysis

Published studies reporting WES data from probands with sporadic autism were examined for de novo mutations in *CACNA1D*. Five studies (24–28) providing data on 980 probands were identified (Table S1 in Supplement 1). Two mutations, p.A749G (A749G; NM_000720 reference sequence) in proband 11872.p1 and p.G407R (G407R) in proband 12620.p1 in the Simons Simplex Collection, were reported in two separate studies (24,25). Both mutations were present in the patient only and not in family members. Both were not reported as variants in the Exome Variant Server (http://evs.gs.washington.edu), the Single Nucleotide Polymorphism Database, and the 1000 Genomes Project. Both were also confirmed as the only *CACNA1D* de novo mutations in a recent WES study including 2303 trios (which included the aforementioned cohort) (29).

### Complementary DNA Constructs

The human wild-type Cav1.3 channel α_1_-subunit (*CACNA1D* gene, National Center for Biotechnology Information reference sequence EU363339, long C-terminal splice variant) containing the alternative exons 8a and 42 was previously cloned into pGFP^minus^ vector (mammalian expression plasmid controlled by cytomegalovirus promoter; it lacks a GFP tag) (30). The A749G and G407R mutations were introduced into the human Cav1.3 construct using standard polymerase chain reaction approaches. Mutated constructs were verified by DNA sequencing (Eurofins MWG Operon; Eurofins Genomics, Ebersberg, Germany).

### Electrophysiologic Recordings in tsA-201 Cells

Cell culture and transient expression of Cav1.3 constructs in tsA-201 cells were performed as described elsewhere (31). Whole-cell patch-clamp recordings were performed at room temperature. Borosilicate glass electrodes were pulled (micropipette puller; Sutter Instrument Company, Novato, California) and fire polished (MF-830 Microforge; Narashige, London, United Kingdom) at a final resistance of 1.5–2.5 MΩ. Cells were recorded at a sampling rate of 2–5 kHz using an Axopatch 200B amplifier (Molecular Devices, Biberach, Germany), digitized with Digitizer 1322A (Molecular Devices), and recorded with pClamp 10.2 software (Molecular Devices). The recording solution contained in mmol/L: bath—15 calcium chloride, 10 *N*-2-hydroxyethylpiperazine-*N*-2-ethanesulfonic acid, 150 choline chloride, and 1 magnesium chloride, adjusted to pH 7.4 with cesium hydroxide; intracellular—135 cesium chloride, 10 *N*-2-hydroxyethylpiperazine-*N*-2-ethanesulfonic acid, 10 cesium–ethylene glycol tetraacetic acid, 1 magnesium chloride, 4 mmol/L disodium adenosine 5′-triphosphate adjusted to pH 7.4 with cesium hydroxide.

Cells were held at a holding potential of −80 mV before a step protocol of 25 msec or 50 msec to different voltages was applied to determine the current-voltage relationship. Currents were leak subtracted using a P/4 protocol. The voltage dependence of inactivation was measured by applying a control test pulse (20 msec to the voltage of maximal inward current [V_max_]) followed by 5-sec conditioning steps to various potentials and a subsequent 20-msec test pulse to V_max_ (30-sec recovery between protocols). Inactivation was calculated as the ratio between the current amplitudes of the test versus control pulse. Estimates for changes in channel open probability or single channel conductance were obtained as described previously (22) by normalizing the ionic tail current after a 20-msec or 25-msec depolarizing pulse to the reversal potential to the “on” gating current (Q_ON_) obtained in the same pulse. Current-voltage curves were fitted to the equation I = G_max_(V − V_rev_)/{1 + exp [− (V − V_.5_)/k]}), where V_rev_ is the reversal potential, V is the test potential, I is the peak current, G_max_ is the maximum conductance, V_.5_ is the half maximal activation voltage, and k is the slope factor. The voltage dependence of calcium conductance was fitted according to a Boltzman distribution G = G_max_/{1 + exp [− (V − V_.5_)/k]}. Steady-state inactivation parameters were obtained by fitting the data to a modified Boltzmann equation: G = (1 − G_max_)/{1 + exp[(V − V_.5_)/k]} + G_max_. To reduce noise in some experiments, protocols were repeated up to five times and recordings were averaged. A junction potential of −9.3 mV was calculated and offline subtracted as previously described (32).

### Immunoblot Analysis

The tsA-201 cells were transfected and cultured as described previously (31). Methods for membrane preparation and immunoblot analysis are described in detail in Supplement 1.

### Statistics

Data analysis was performed using pClampfit 10.2 (Molecular Devices), SigmaPlot 12 (Systat Software GmbH, Erkrath, Germany) or GraphPad Prism 5.1 software (GraphPad Software, Inc, La Jolla, California). All values are presented as mean ± SEM for the indicated number of experiments (*n*) except if stated otherwise. Data were analyzed by unpaired Student *t* test, Mann-Whitney test, and one-way analysis of variance followed by Bonferroni posttest or Kruskal-Wallis test followed by Dunnʼs posttest as indicated for individual experiments. Statistical significance was set at *p* < .05.

## Results

### *CACNA1D* De Novo Mutations in WES Studies of Patients with Autism

The two identified *CACNA1D* de novo mutations, A749G in proband 11872.p1 and G407R in proband 12620.p1, were absent in the parents (family 11872) (24) and in the parents and an unaffected sibling (family 12620) (25). Only A749G was classified as a high-risk ASD mutation in the original publication (24). In both probands, A749G was the only amino acid–changing mutation. In patient 11872.p1, another risk mutation of unknown functional relevance (splice site mutation in *KATNAL2*) was reported (24). In proband 12620.p1, a synonymous mutation in *ADAMTSL1* was found. No large copy number variations were reported in the two probands. No other major phenotypes (e.g., seizure, hyperaldosteronism) were reported (24,25). Nonverbal IQ was 65 for proband 11872.p1 (24).

### Biophysical Properties of A749G and G407R

**Figure 1:**
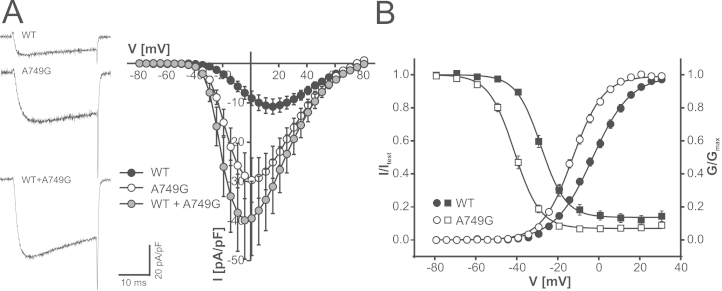
Biophysical properties of A749G expressed in tsA-201 cells. **(A)** Calcium current voltage relationships for human wild-type and A749G and A749G mutants coexpressed together with wild-type (WT + A749G, equal amounts of complementary DNA transfected for both constructs) in tsA-201 cells as described in Methods and Materials. Sample traces of inward calcium currents measured during depolarizations to maximum voltage are also shown. Current-voltage curves include only data for wild-type channels pooled from parallel recordings with mutants in the same transfections (six independent transfections) to account for differences in expression levels between transfections. A749G cotransfected with wild-type (WT + A749G) resulted in significantly increased peak current amplitudes (for statistics and numbers, see Results). Statistics for gating parameters are summarized in Table 1. **(B)** Steady-state activation (circles) and inactivation (squares) curves for wild-type and A749G were obtained as described in Methods and Materials. Means ± SEM are illustrated. Wild-type, *n* = 29 (nine transfections); A749G, *n* = 27 (six transfections). Steady-state activation parameters for WT + A749G are given in Table 1.

**Figure 2:**
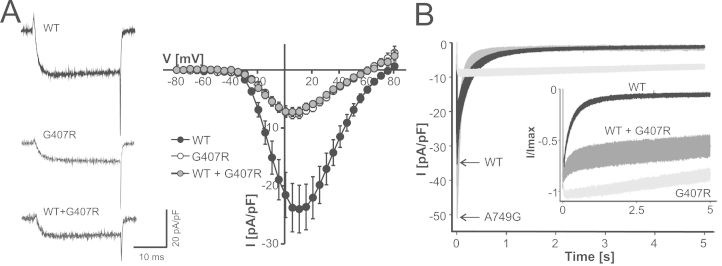
Functional consequences of A749G and G407R expressed in tsA-201 cells. **(A)** Calcium current voltage relationships for human wild-type and G407R and G407R mutants coexpressed together with wild-type (WT + G407R, equal amounts of complementary DNA transfected for both constructs) in tsA-201 cells as described in Methods and Materials. Sample traces of inward calcium currents measured during depolarizations to maximum voltage are also shown. Current-voltage curves include only data for wild-type channels pooled from parallel recordings with mutants in the same transfections (*n* = 4) to account for differences in expression levels between experiments. Transfections with high expression levels of wild-type were selected for analysis to provide sufficient current amplitudes for G407R channels. Peak current amplitudes of G407R mutants and G407R cotransfected with wild-type were significantly reduced (mean ± SEM [pA/pF]; wild-type, −24.6 ± 4.18, *n* = 15; G407R, −8.12 ± .70, *n* = 13; WT + G407R, −7.63 ± .87, *n* = 9; both *p* < .0001 vs. wild-type, Mann-Whitney test). Statistics for gating parameters obtained from all transfections are summarized in Table 1.**(B)** Inactivation of wild-type, A749G, and G407R mutants during 5-sec depolarizations from a holding potential of −80 mV to maximum voltage. Peak inward calcium currents for overlapping wild-type and A749G traces are indicated by arrows for clarity. Traces represent the means ± SEM (only ± SEM is illustrated) from parallel recordings of wild-type and mutant channels performed on the same day in four (G407R) or two (A749G) independent transfections. The pronounced slowing of inactivation is evident from the respective normalized current traces shown in the inset. See text for numbers and % current persisting after 5-sec depolarizations. WT, wild-type.

Data from previous mutational studies in Cav1 α_1_-subunits (33,34) strongly suggested interference of both mutations with Cav1.3 LTCC function. G407R (present in exon 8a, one of two alternative exons) is identical to a Timothy syndrome mutation in Cav1.2 α_1_-subunits (17). In addition, A749G is located adjacent to p.I750M (I750M), a Cav1.3 mutation for which we reported a pronounced gain-of-function (22). Analysis of the two mutations in a homology model of the Cav1.3 α_1_-subunit also predicted pronounced changes of the interaction of the affected distal S6 helices with adjacent S6 helices of the activation gate (G407R, A749G) and of the S4–S5 linker with the voltage sensor (G407R) (Figure S1 in Supplement 1). This analysis prompted us to introduce both mutations into human Cav1.3 α_1_-subunits and express them in tsA-201 cells together with α_2_δ_1_ and β_3_ accessory subunits, which form most LTCC complexes in the brain (35). Western blots revealed robust expression of intact α_1_-subunits, although slightly higher and lower expression of the G407R and A749G mutant proteins were observed, respectively (Figure S2 in Supplement 1). Both mutations strongly affected channel gating (Figures 1 and 2). A749G significantly enhanced peak current amplitudes (wild-type, −11.6 ± 2.2 pA/pF, *n* = 17; A749G, −30.5 ± 5.8 pA/pF, *n* = 27; *p* < .0001, Mann-Whitney test; wild-type controls from same transfection experiments) (Figure 1A); this was not due to an increased surface expression estimated by quantification of the Q_ON_, which was significantly decreased in the mutant (Q_ON_ [pA*ms] wild-type, 170 ± 26.8, *n* = 28; A749G, 80.5 ± 11.4, *n* = 20; *p* < .009). A749G also shifted steady-state activation and inactivation voltage dependence (Figure 1B) of inward calcium currents to more negative potentials (Table 1).

Depolarizations to the reversal potential revealed an increased ratio of maximal tail current amplitude to integrated Q_ON_ (I_tail_/Q_ON_ [msec^−1^]: wild-type, 11.1 ± 1.1, *n* = 28; A749G, 34.1 ± 2.25, *n* = 20; *p* < .0001 vs. wild-type, unpaired Student *t* test). This finding is compatible with a higher channel open probability or conductance or both, a feature previously observed by us also for other Cav1.3 gain-of-function mutations (22). In contrast to A749G, mutation G407R reduced maximal current amplitudes (Figure 2A) and caused no change in activation voltage dependence (Figure 2A and Table 1). However, in contrast to A749G, G407R dramatically slowed the inactivation time course during 5-sec depolarizations. At the end of the 5-sec pulse to V_max_, only 5.12% ± .98% (*n* = 15) of maximal wild-type and 3.07% ± .37% (*n* = 6) of A749G current remained, whereas 82.8% ± .04% (*n* = 13) of G407R current persisted (*p* < .001 vs. wild-type, Mann-Whitney test) (Figure 2B, inset). The failure of G407R currents to inactivate prevented the measurement of steady-state inactivation parameters. Despite reduced maximal current amplitudes (Figure 2A), the slow inactivation resulted in larger absolute current amplitudes during prolonged depolarization than in wild-type Cav1.3 channels (Figure 2B). The absence of a fast inactivating component (mediated by calcium-dependent inactivation in Cav1.3) (9,36) suggests that calcium-dependent and voltage-dependent inactivation were strongly weakened by the mutation. The smaller current and Q_ON_ amplitudes were unlikely because of a lower expression of mutant channel protein as demonstrated by Western blots (Figure S2 in Supplement 1).

Because patients are heterozygous for both mutations, we next tested if the gain-of-function phenotype is also maintained when wild-type channels are coexpressed with the mutants by transfection of equal quantities of α_1_-subunit cDNAs; as evident from Figures 1 and 2 and Table 1, this was the case. For coexpression of wild-type with A749G, the gain-of-function was driven by the much larger current amplitude of the mutant shifting overall steady-state activation to more negative voltages, such as for A749G alone. Combined expression yielded an I_tail_/Q_ON_ ratio in between wild-type and A749G values (I_tail_/Q_ON_ [msec^−1^]: wild-type + A749G, 22.2 ± 2.8, *n* = 11; *p* < .01 vs. wild-type or vs. A749G, one-way analysis of variance, Bonferroni posttest). This finding is consistent with an equal contribution of the two constructs to tail currents. In cells transfected with wild-type and the G407R mutant, current amplitude was reduced similar to G407R expression alone, and a slowly inactivating component dominated overall current kinetics (Figure 2B, inset). Similar to for G407R expressed alone, a large fraction of inward calcium currents remained at the end of a 5-sec pulse to V_max_ (55.7 ± 8.46, *n* = 10; *p* < .05 vs. wild-type, not significant vs. G407R alone, Kruskal-Wallis test, Dunnʼs posttest). Reduced current amplitude was unlikely because of reduced α_1_-subunit protein expression (Figure S2 in Supplement 1). Compared with wild-type, this mutant also induced enhanced calcium transients when expressed in electrically excitable GLT-myotubes (Figure S3 in Supplement 1). Taken together, our data demonstrate that both mutations increase Cav1.3 channel activity even when expressed in the heterozygous state.

## Discussion

Our novel functional data strongly argue for an important (perhaps causal) role of Cav1.3 in the pathophysiology of ASD. Two de novo missense mutations were reported among 2303 trios, showing *CACNA1D* as one of the genes with recurrent mutations in ASD. We found that both mutations affect evolutionary highly conserved regions in the channelʼs activation gate and disrupt normal channel activity by inducing a pronounced gain of channel function. This potential pathogenetic role of Cav1.3 hyperactivity for brain disease is further strengthened by previous finding of two other Cav1.3 channel-activating mutations (e.g., I750M; see further on) reported to cause PASNA, a severe syndrome with neurodevelopmental deficits and seizures at an early age (22,23). One of the mutations investigated here, G407R, is identical to G403R in related Cav1.2 channels that causes Timothy syndrome. Most patients with Timothy syndrome also have autism (17), and mice containing the human mutation replicate autistic behavioral traits (18). In mice, short-term pharmacologic activation of Cav1.3 induces depression-like behaviors; this is also compatible with a role of this channel in neuropsychiatric symptoms (8). Cav1.3 LTCCs have been shown to play a crucial role in synapse formation and dendritic refinement in mice (37,38). Because many molecules whose mutations or polymorphisms are associated with ASD play a role in neuronal development, neuronal or synaptic differentiation, or synaptic signaling (39), Cav1.3 channels can be regarded as another member of the postsynaptic signaling cascade found to be affected in patients with ASD. This idea is further supported by the fact that Cav1.3 forms direct interactions with shank, a protein well known to play a pathogenic role in ASD (39–41).

The two germline mutations previously identified in the two patients with PASNA raise the clinically relevant question about why such symptoms (including aldosteronism and epilepsy) were not reported in the two patients with ASD described in this study. Although the number of mutations for an extensive genotype-phenotype correlation is not yet large enough, it is possible that different activating *CACNA1D* mutations can induce a disease spectrum of different clinical manifestations, as reported for mutations in Cav2.1 (*CACNA1A*) (42). It can be predicted that the mutational consequences on channel function strongly depend on the firing pattern of neurons. For example, a shift of steady-state inactivation to more negative voltages may reduce the channel availability in neurons with depolarized membrane potentials, such as dopamine neurons in the substantia nigra or ventral tegmental area. This shift may also reduce the impact of other gating changes promoting calcium influx. Mutant mice expressing Cav1.3 gain-of-function mutations are required to confirm this hypothesis.

Mutations can increase Cav1.3 calcium current activity through different biophysical mechanisms as exemplified by the two mutants studied here and in somatic gain-of-function mutations characterized in aldosterone-producing adenomas (22,23). On the macroscopic current level, they can induce channel activation and inactivation gating at more negative voltages, such as A749G, or reduce channel inactivation during depolarizing stimuli, such as G407R (reported here) or P1336R (23). I750M is located adjacent to A749G and was one of the mutations causing PASNA (23). I750M channels combine the features of A749G and G407R because I750M not only displays a more negative activation and inactivation voltage range (similar to A749G) but also strongly slows inactivation (similar to G407R) and does not decrease overall current amplitude (23). This stronger functional change could easily explain its more severe clinical consequences in patients with PASNA; this is further supported by the similar biophysical changes observed for the other PASNA mutation, G403D (23). One could envisage that “mild” Cav1.3 gain-of-function (as proposed in the two mutations analyzed here) causes or strongly predisposes to the development of ASD, whereas “strong” gain-of-function leads to PASNA with aldosteronism, severe neurodevelopmental disturbances, and seizures manifesting soon after birth.

In conclusion, our findings have immediate relevance for clinical practice. In contrast to Cav1.2, Cav1.3 is not expressed in ventricular cardiomyocytes (43). Cav1.3 activating mutations are not expected to cause long QT syndrome, a frequent cause of early cardiac death in patients with Timothy syndrome (17). Because Cav1.3 is expressed in the sinoatrial node and atrial myocytes, supraventricular cardiac symptoms may occur in carriers of activating Cav1.3 mutations. These may also cause primary aldosteronism and hypertension with or without hyperkalemia (23). Taken together, these data provide a strong rationale for close monitoring of the two patients with ASD for such symptoms throughout life. Clinical studies in humans have shown that most available dihydropyridine LTCC blockers used to treat hypertension also permeate the blood-brain barrier and can affect neuronal plasticity (44). Similar to Cav1.3 deficiency in mice, this does not cause unwanted central nervous system side effects in treated individuals. Safety has been confirmed in a phase II study with isradipine (which, similar to other dihydropyridines, blocks both Cav1.2 and Cav1.3 channels) in preparation for ongoing trials for neuroprotection in patients with Parkinson’s disease (phase III, ClinicalTrials.gov Identifier: NCT02168842) and as adjunct therapy in patients with bipolar depression (phase II, ClinicalTrials.gov Identifier: NCT01784666). Inhibition of LTCCs in the brain may ameliorate psychiatric symptoms in the carriers of Cav1.3-activating *CACNA1D* mutations identified in our study. Given the excellent toxicity profile of dihydropyridine LTCC blockers, long-term off-label treatment of patients with ASD appears justified based on our robust in vitro findings. Given enhanced depression-like behaviors in mice after selective Cav1.3 LTCC activation (8,21), *CACNA1D* should also be examined as a possible risk gene for depression and anxiety disorders in humans.

## Figures and Tables

**Table 1:** Gating Properties of Cav1.3 α_1_-Subunit (*CACNA1D*) Mutations A749G and G407R Compared with Wild-Type Cav1.3 Channels

α_1_ Subunit	Activation				Steady-State Inactivation			
	V_.5_ (mV)	Slope (mV)	V_rev_ (mV)	*n*	V_.5_ (mV)	Slope (mV)	Noninactivating (%)	*n*
WT	−2.55 ± 1.05	8.92 ± .20	67.7 ± 1.14	29	−25.7 ± 2.08	5.56 ± .23	14.4 ± 3.12	18
A749G	−12.3 ± .87^a^	7.14 ± .20^a^	60.3 ± .83^a^	27	−41.1 ± 1.07^a^	5.82 ± .18	9.43 ± 1.49	14
WT + A749G	−15.0 ± 1.71^a^	6.52 ± .40^a^	59.8 ± 1.66^b^	11	ND			
G407R	−6.58 ± 1.41	7.90 ± .30	54.7 ± 2.32^a^	13	ND			
WT + G407R	−8.15 ± 2.00	8.07 ± .82	58.9 ± 2.99^b^	9	ND			

All values are mean ± SE. Number of independent transfections was ≥2. Parameters were obtained after fitting normalized (I/I_max_) I–V relationships or normalized steady-state inactivation curves as described in Methods and Materials. Statistical analysis was performed using one-way analysis of variance and Bonferroni post hoc test (activation) or unpaired Student *t* test (steady-state inactivation) as indicated. *n*, number of experiments; ND, not determined; V_.5_, half maximal activation/inactivation voltage; V_rev_, reversal potential; WT, wild-type.

a *p* < .001 compared with WT.

b *p* < .01 compared with WT.
