# Supplementary material for: CACNA1D De Novo Mutations in Autism Spectrum Disorders Activate Cav1.3 L-Type Calcium Channels
Source: Biol Psychiatry. 2015 May 1;77(9):816–22. doi: 10.1016/j.biopsych.2014.11.020 (PMC4401440; doi:10.1016/j.biopsych.2014.11.020)
Supplement: Supplementary file 1 — Supplementary Material [file mmc1.pdf]

CACNA1D De Novo Mutations in Autism Spectrum Disorders Activate Cav1.3 L-type Ca^{2+} Channels

Supplemental Information

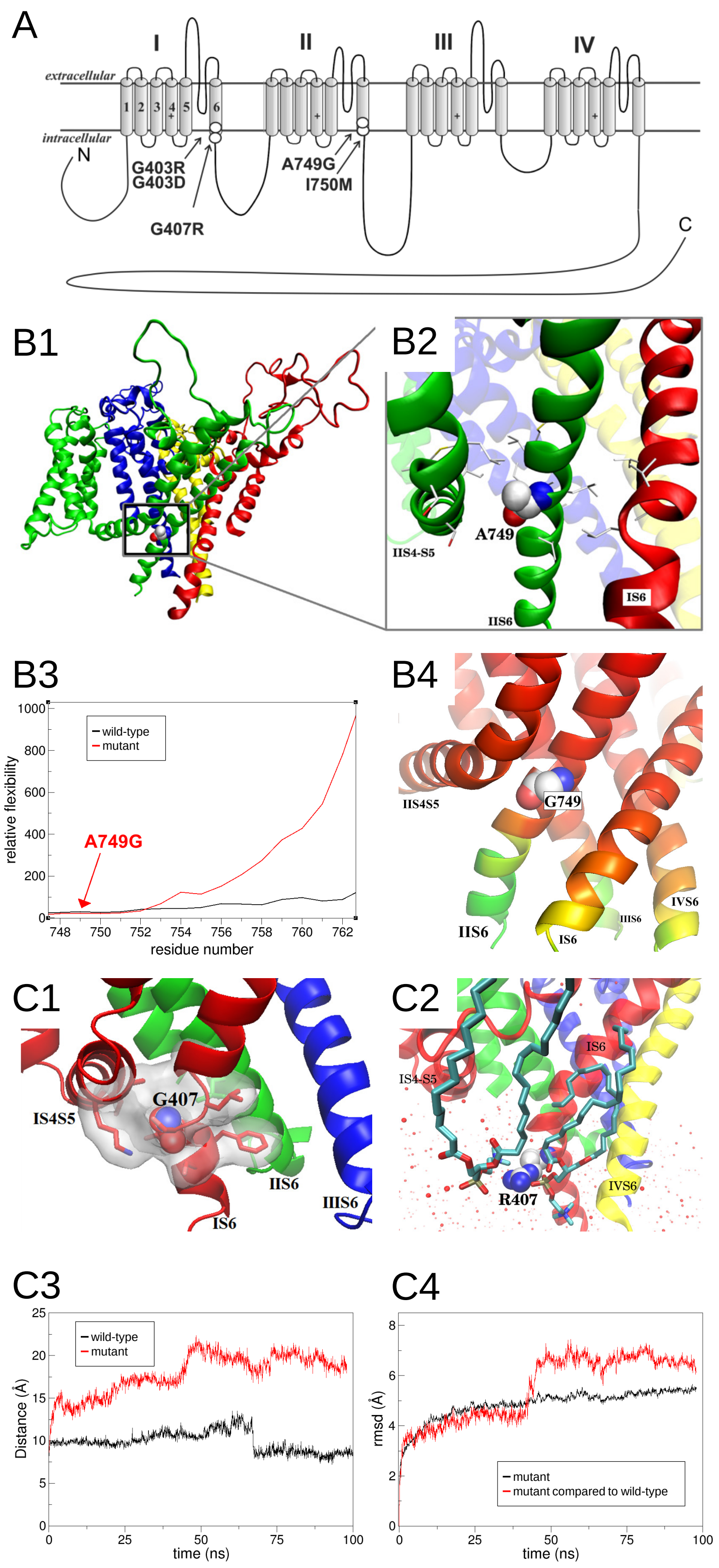

Figure S1. Position of the Cav1.3 mutations A749G and G407R within the pore forming Cav1.3 a1-subunit.

A. Simplified scheme of Cav1.3 a1-topology with its four homologous repeats (segments S1 – S6). A749G and G407R (in alternative exon 8A) are shown together with two missense mutations discovered in aldosterone producing adenomas (somatic mutations G403R and I750M) for which a gain of function phenotype has been confirmed in previous functional studies (1, 2). Mutations G403D (in alternative exon 8B) and I750M were also reported as germline mutations in individuals exhibiting congenital neurodevelopmental defects, intellectual disability and epilepsy in addition to hyperaldosteronism (PASNA) (2).

B. Prediction of structural changes imposed by mutation A749G: **B1.** Side view of the ion pore (S5, S6 and connecting linkers of all four domains). Homologous repeats are shown in different colors (I, red; II, green; III; blue; IV, yellow). For clarity only the voltage sensor (IIS1-IIS4) of repeat II (green) is shown. **B2.** Ala749 (A749) is located in the activation gate (cytoplasmic ends of each S6) of repeat II. As expected (3), in the closed state our model predicts contacts with the IIS4-IIS5 linker which connects the activation gate with the voltage-sensing domain of this repeat. In wild-type channels these interactions are stable during MD simulations. **B3.** However, 100-ns MD simulations of mutant A749G revealed a strong increase in the flexibility of the IIS6 helix distal to residue 749 relative to wild-type. **B4.** The higher flexibility of the distal IIS6 helix is illustrated by color coding (low B-factor in red, high B-factor in green). This also results in a higher probability of IIS6 interactions with the adjacent S6 helices as part of the activation gate (not illustrated). Methodological details on modeling and MD simulations are given in the Supplemental Methods.

C. Prediction of structural changes imposed by mutation G407R: Location of residue 407 in the wild-type (C1) and mutant (C2) Cav1.3 a1-subunit. In the wild-type channel, it makes close contacts to the S4-S5 linker of repeat I (in particular residues S263, K266, A267). This is illustrated by the Van der Waals surface (grey) in **C1.** Panel **C2** clearly shows that the IS4-IS5 interactions predicted in the wild-type are completely lost in the mutant channel. Moreover, the introduction of a positive charge enables new electrostatic interactions with adjacent IIS6 and IVS6 helices as part of the activation gate (not illustrated). The model also predicts that R407 can form ionic interactions with charged heads of lipids which additionally indicates the loss of interactions with the S4-S5-linker in the mutant. This is also demonstrated by the plot in **C3**, showing the distance between the IS6 activation gate and the IS4S5 linker in the wild-type (black line) and the mutant (red line). In wild-type the distance remains stable at about 10 Å and even decreases during the simulation. In contrast, the mutant G407R shows an increase of the distance up to 20 Å. Thus, the activation gate of domain I is moving away from the IS4S5 linker and is expected to lose key interactions for coupling with its voltage sensor. This is also confirmed by the root mean square deviation (RMSD) of the mutant with respect to the wild-type channel as shown

in **C4.** The RMSD plot of G407R indicates that the structural model is stable after equilibration (indicated by the black line) and differs from the wild-type conformation (RMSD increases up to 7 Å, indicated by the red line).

Although the predictions of our molecular model still need to be confirmed by further mutational analysis, they clearly demonstrate that the mutations occur within regions that are crucial for the electromechanical coupling of the voltage-sensing domains to the channel pore and normal function of the activation gate (for recent reviews see 4, 5).

The slowing of the inactivation in G407R can be explained by a loss of Ca^{2+}-dependent inactivation and a slowing of voltage-dependent inactivation, because inactivation is much slower than observed with barium currents through Cav1.3 wild-type channels (cf. 6). This highlights the importance of the IS6 helix also for voltage-dependent inactivation of Cav1.3 channels. The mobility of the IS6 segment can also be affected by Ca^{2+} channel b-subunits through binding to the I-II-linker of the channel (7-9) and also allows control of voltage-dependent inactivation (10). It will therefore be important to analyze Cav1.3 gain of function mutations also with b-subunit isoforms other than b3 in future studies.

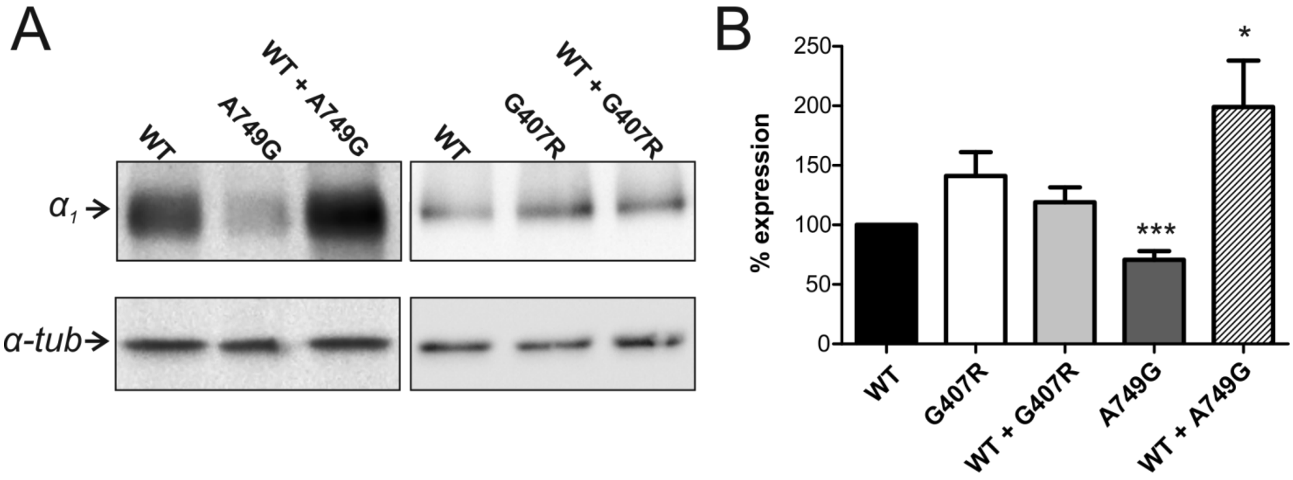

Figure S2. Expression of Cav1.3 a1-subunit immunoreactivity in tsA-201 cells.

A. Western blots from at least two independent membrane preparations of transfected tsA-cells were carried out as described in Methods. For quantification, immunoreactivities of individual α1-subunits were normalized to α-tubulin (α-tub) immunoreactivity as loading control. **B.** Quantification of expression levels normalized to wild-type a1 (% of WT, means ± S.E.M.; A749G: 70.74 ± 7.24, *n* = 17, A749G co-expressed with WT: 199.2 ± 38.9, *n* = 7, G407R: 141.1 ± 20.1, *n* = 5, G407R co-expressed with WT: 119.1 ± 12.4, *n* = 5). ****p* = 0.0009 against WT; **p* = 0.0436 against WT, one-sample *t*-test. The lower protein expression of A749G in comparison to wild-type is likely due to our finding (several independent transfections) of a lower number of transfected cells surviving in the culture in comparison to wild-type compatible with mutation-induced Ca^{2+} - toxicity.

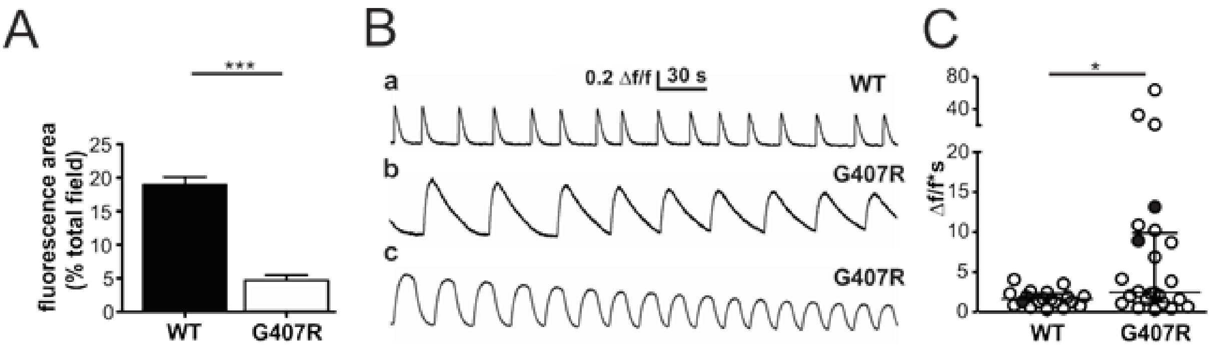

Figure S3. G407R increases Ca^{2+} oscillations in dysgenic myotubes.
To confirm that the smaller maximal current size measured for G407R can indeed affect excitability in an electrically excitable cell, we expressed this mutant in skeletal muscle GLT-myotubes. These lack functional a1-subunits, express native auxiliary b- and a2d-subunits and thus allow current recordings that are not contaminated by other Ca^{2+} channels.

A. Area of fluorescent signal (% of total field) from GLT myotubes expressing WT (n = 16) or G407R (n = 21). A significantly smaller area ($p < 0.0001$, Mann-Whitney test) is measured in presence of G407R. This reflects a smaller size as well as a smaller number of myotubes expressing G407R in comparison to those expressing WT channels. Together with signs of reduced development observed during the course of these experiments this might imply cellular toxicity. **B.** Ca^{2+} imaging: fluorescence emission from myotubes loaded with Fluo-4 reveals spontaneous Ca^{2+} oscillations in samples transfected with wild-type (n = 19 out of 28) or G407R mutant channels (n = 24 out of 28) but not in non-transfected GLT cells (n = 5, not shown). Example traces for events larger than wild-type events from randomly selected mCherry (transfection marker) positive cells are shown. **C.** Integral of single Ca^{2+} oscillations over time (Δf/f*s) in randomly selected myotubes expressing WT (n = 19) or G407R mutant (n = 24) channels. Filled circles represent the data from the sample traces shown in panel B. Notice the break in the Y-axis. Although amplitudes ($p = 0.25$) and frequency ($p = 0.83$) of spontaneous Ca^{2+} oscillations were not significantly different in G407R-transfected GLTs compared to wild-type, a larger area underneath spontaneous oscillations was measured in a significant proportion of cells expressing G407R (Δf/f*s > 5, n = 9 out of 24) which were absent in GLT cells transfected with wild-type Cav1.3 (Δf/f*s > 5 n = 0 out of 19). These changes involved a longer duration of the single events due to a slower Δf/f decay (example trace b) and/or to a longer plateau at each event peak (example trace c). In different myotubes these changes contributed to varying degrees to the shape of the larger single events. Taken together, these data further support a gain-of-function phenotype for the G407R mutation despite its smaller maximal current amplitudes in transfected cells.

Table S1. Publications reporting whole-exome sequencing data for *de novo* mutations in ASD patients. Amino acid positions refer to reference sequence NM_000720.

Study	Number of Families	*CACNA1D* Mutation (proband)	Coordinates (hg19)	Reference
Iossifov *et al.* (2012)	343 (quads, Simon Simplex Collection)	p.G407R (12620.p1)	chr3:53707152	11
O'Roak *et al.* (2012)	189 (trios)	p.A749G (11872.p1)	chr3:53764493	12
Sanders *et al.* (2012)	225 (quads or trios, Simon Simplex Collection)	not reported	not reported	13
Neale *et al.* (2012)	175 (trios, 6 centers)	not reported	not reported	14
An *et al.* (2014)	48 (trios and sibling families)	not reported	not reported	15

The two de novo events have been recently confirmed in another study including the cohort with the two patients (16).

Supplemental Methods

Immunoblot Analysis

tsA 201-cells were transfected and cultured as described previously (17). Membrane preparations were performed 3 days after transfection. Cells were washed with phosphate buffered saline (in mM: 137 NaCl, 2.7 KCl, 8 Na_2HPO_4, 1.5 KH_2PO_4), harvested and resuspended in 2 ml lysis buffer (10 mM Tris-HCl, 1 µg/ml aprotinin, 0.1 mg/ml trypsin inhibitor, 1 µM pepstatin A, 0.5 mM benzamidine, 0.2 mM phenylmethylsulfonylfluoride, 2 mM iodacetamide, 1 µl/ml leupeptin, pH 7.4) and lysed on ice for 15 minutes. The mixture was then resuspended, homogenized by forcing it through a cannula (27 G) and centrifuged for 20 min at 726 x g. Membranes were collected from the resulting supernatant by ultracentrifugation at 110,561 x *g* for 30 min. The resulting pellet was resuspended in 150-200 µl of lysis buffer and stored at -80°C. α1 subunits were detected using affinity-purified rabbit anti-Cav1.3 α1-subunit antibody (directed against amino acids 2022-2138, NCBI reference sequence M76558; 18). Anti α-tubulin (mouse-monoclonal (DM1A), CAT: CP06, emd Millipore, diluted 1:100,000) was used as loading control. Peroxidase conjugated goat anti-rabbit IgG (1:40,000, whole molecule Sigma A0545, Lot #038K4753) and goat anti-mouse IgG (1:10,000, Prod # 31430, Lot # EG767118) were used as secondary antibodies. Signals were detected with the image acquisition system Fx7 (Peqlab). Quantitation of band intensity was performed using ImageJ 1.46 (National Institute of Health). After background subtraction integrated densities of mutant and wild-type signals were normalized to the loading control.

Molecular Modeling

Since 3D-structural information from crystallography is not yet available for Cav1.3 a1-subunits, we predicted the structure of the wild-type and conformational changes induced by the mutations by developing a homology model based on the Ca^{2+}-selective NavAb channel in the inactivated state (PDB code 4MVZ; (3)). Homology modeling has been performed

using MOE (Molecular Operating Environment, version 2013.08, Molecular Computing Group Inc., Montreal, Canada). We took advantage of the high sequence conservation (~60% similarity and ~35% identity measured by MOE) in the transmembrane segments of Cav1.3 a1 and NavAb to build a model for the pore-forming regions of Cav1.3. Sequences were aligned with ClustalW, as already published by Zhang and coworkers for NavAb and Cav1.3 (19). However, sequences are not conserved in loops. Therefore *ab initio* modeling has to be used to generate stable conformations taking into account only the sequence information. In particular, we used the Rosetta method to build models for loops (20, 21). Moreover, we used the *ab initio* Rosetta method to generate structures for the activation gates that were missing in the NavAb channel template. In the case of mutations, the structure was derived from the wild-type model by replacing the mutated residue and carrying out a local energy minimization of its atoms using dedicated tools of MOE. Moreover, in each case the C-terminal and N-terminal parts of each domain were capped to avoid perturbations by free charged functional groups. Once the protein model was completed, it was embedded in the virtual plasma membrane containing POPC (1-palmitoyl-2-oleoyl-sn-glycero-3-phosphocholine) and cholesterol in a 3:1 ratio, using the CHARMM-GUI Membrane Builder (22). It included also water molecules and $CaCl_2$ (150 mM) in the simulation box. Subsequently, energy minimization in membrane environment was performed with starting coordinates and topology generated by LeaP Amber14 tool (23), using dedicated force fields for proteins and lipids, ff14SBonlysc and Lipid14 respectively (24). The system was gradually heated from 0 to 300 K in two steps, keeping the lipids fixed, and then equilibrated over 1 ns. Then MD simulations were performed for 100 ns, with time steps of 2 fs, at 300 K and in anisotropic pressure scaling conditions that is suitable for membrane proteins. Van der Waals and short-range electrostatic interactions were cut off at 10 Å, whereas long-range electrostatics were calculated by the Particle Mesh Ewald (PME) method. Trajectories were analyzed by CPPTRAJ Amber14 tool (23) and visualized using VMD (version 1.9.1, 25). Moreover, the identification of the key interactions was aided by other visualization tools, such as MOE and Pymol Molecular Graphics System (version

1.6.0.0, Schrödinger, LLC). The analyses of both mutations resulted in detailed quantitative data, such as the relative flexibility and distances between specific residues. Furthermore, the quality of the models has been evaluated using experimental knowledge (also from other Ca^{2+} channel isoforms) and molecular dynamics simulations on our in-house GPU cluster that permitted to perform fast calculations (26). Parameters like the root mean square deviation were investigated to determine the structural stability of the models after minimization in their natural lipid environment.

Electrophysiological Recordings and Ca^{2+} Imaging in Dysgenic Myotubes

Myotubes of the homozygous dysgenic (mdg/mdg) cell line GLT lack functional a1-subunits and express native auxiliary b- and a2d-subunits and thus allow current recordings that are not contaminated by other Ca^{2+} channels. Myotubes were cultured and transfected as previously described (27) and analyzed 4 days after transfection. Cells transfected with Cav1.3 wild-type a1 or Cav1.3 G407R together with eGFP were used in parallel for live cell imaging and electrophysiological recordings. Images were acquired with a digital camera (Canon) mounted at an inverted microscope (Zeiss). Non-corrected images were analyzed with ImageJ. The fluorescence area (% of total field) was measured after binarization and thresholding images at 50 of 256 bits. For electrophysiological measurements the patch pipettes (borosilicate glass, Harvard Apparatus, Holliston, MA) had a resistance of 2.5–3.5 MΩ when filled with (in mM) 150 N-methyl-D-glucamine, 10 EGTA, 1 $MgCl_2$ 10 HEPES, 4 Mg-ATP. pH was adjusted to 7.3 with methansulfonate; the bath solution contained (in mM): 10 $CaCl_2$, 145 tetraethylammoniumchloride, 10 HEPES (pH 7.4 with tetraethylammoniumhydroxide).

For Ca^{2+} imaging experiments myotubes were co-transfected with Cav1.3 wild-type a1 or Cav1.3 G407R and m-Cherry. Red-fluorescent myotubes were targeted for imaging. Cells were kept in tyrode solution containing (in mM): 130 NaCl, 2.5 KCl, 2 $CaCl_2$, 2 MgCl2, 10 HEPES, 30 glucose, 0.2% bovine serum albumin (pH 7.4 with NaOH). To load the Ca^{2+}

indicator, GLT myotubes were incubated for 45 minutes in Tyrode-solution with 0.2 mM Fluo-4 AM (Invitrogen), and 2 µM pluronic acid. Experiments were carried out at room temperature (21-23°C). The fluorescent signal from single myotubes was acquired through photomultipliers with the software Felix (Photon Technology International). Data were processed in Microsoft Excel and analyzed with Clampfit (Axon Instruments). Fluorescent emission from each sample was monitored for 10 minutes. Repetitive oscillations within each given cell showed virtually identical kinetics. However during 10 minutes-recording, a variable amount of bleaching was observed. The analysis of Ca^{2+} oscillations (except the frequency) was therefore carried out on single events at the beginning of each trace (typically the first or second complete event). Fluorescent oscillations were normalized to baseline (ΔF/F).

Supplemental References

1. Azizan EA, Poulsen H, Tuluc P, Zhou J, Clausen MV, Lieb A, *et al.* (2013): Somatic mutations in ATP1A1 and CACNA1D underlie a common subtype of adrenal hypertension. *Nat Genet*. 45:1055-1060.

2. Scholl UI, Goh G, Stolting G, de Oliveira RC, Choi M, Overton JD, *et al.* (2013): Somatic and germline CACNA1D calcium channel mutations in aldosterone-producing adenomas and primary aldosteronism. *Nat Genet*. 45:1050-1054.

3. Tang L, Gamal El-Din TM, Payandeh J, Martinez GQ, Heard TM, Scheuer T, *et al.* (2014): Structural basis for Ca2+ selectivity of a voltage-gated calcium channel. *Nature*. 505:56-61.

4. Blunck R, Batulan Z, Phillips LR, Milescu M, Li-Smerin Y, Mindell JA, *et al.* (2012): Mechanism of electromechanical coupling in voltage-gated potassium channels. *Front Pharmacol.* 3:166.

5. Catterall WA (2014): Structure and function of voltage-gated sodium channels at atomic resolution. *Exp Physiol*. 99:35-51.

6. Bock G, Gebhart M, Scharinger A, Jangsangthong W, Busquet P, Poggiani C, *et al.* (2011): Functional properties of a newly identified C-terminal splice variant of Cav1.3 L-type Ca^{2+} channels. *J Biol Chem*. 286:42736-42748.

7. Dolphin AC (2012): Calcium channel auxiliary alpha2delta and beta subunits: trafficking and one step beyond. *Nat Rev Neurosci*. 13:542-555.

8. Neely A, Hidalgo P (2014): Structure-function of proteins interacting with the alpha1 pore-forming subunit of high-voltage-activated calcium channels. *Front Physiol.* 5:209.

9. Vitko I, Shcheglovitov A, Baumgart JP, Arias O, II, Murbartian J, Arias JM, *et al.* (2008): Orientation of the calcium channel beta relative to the alpha(1)2.2 subunit is critical for its regulation of channel activity. *PLoS One*. 3:e3560.

10. Koschak A, Reimer D, Huber I, Grabner M, Glossmann H, Engel J, *et al.* (2001): a1D (Cav1.3) subunits can form L-type calcium channels activating at negative voltages. *J Biol Chem*. 276:22100-22106.

11. Iossifov I, Ronemus M, Levy D, Wang Z, Hakker I, Rosenbaum J, *et al.* (2012): De novo gene disruptions in children on the autistic spectrum. *Neuron*. 74:285-299.

12. O'Roak BJ, Vives L, Girirajan S, Karakoc E, Krumm N, Coe BP, *et al.* (2012): Sporadic autism exomes reveal a highly interconnected protein network of de novo mutations. *Nature*. 485:246-250.

13. Sanders SJ, Murtha MT, Gupta AR, Murdoch JD, Raubeson MJ, Willsey AJ, *et al.* (2012): De novo mutations revealed by whole-exome sequencing are strongly associated with autism. *Nature*. 485:237-241.

14. Neale BM, Kou Y, Liu L, Ma'ayan A, Samocha KE, Sabo A, *et al.* (2012): Patterns and rates of exonic de novo mutations in autism spectrum disorders. *Nature*. 485:242-245.

15. An JY, Cristino AS, Zhao Q, Edson J, Williams SM, Ravine D, *et al.* (2014): Towards a molecular characterization of autism spectrum disorders: an exome sequencing and systems approach. *Transl Psychiatry*. 4:e394.

16. De Rubeis S, He X, Goldberg AP, Poultney CS, Samocha K, Ercument Cicek A, *et al.* (2014): Synaptic, transcriptional and chromatin genes disrupted in autism. *Nature*.

17. Ortner NJ, Bock G, Vandael DH, Mauersberger R, Draheim HJ, Gust R, *et al.* (2014): Pyrimidine-2,4,6-triones are a new class of voltage-gated L-type Ca^{2+} channel activators. *Nat Commun.* 5:3897.

18. Platzer J, Engel J, Schrott-Fischer A, Stephan K, Bova S, Chen H, *et al.* (2000): Congenital deafness and sinoatrial node dysfunction in mice lacking class D L-type calcium channels. *Cell*. 102:89-97.

19. Zhang X, Ren W, DeCaen P, Yan C, Tao X, Tang L, *et al.* (2012): Crystal structure of an orthologue of the NaChBac voltage-gated sodium channel. *Nature*. 486:130-134.

20. Yarov-Yarovoy V, Baker D, Catterall WA (2006): Voltage sensor conformations in the open and closed states in ROSETTA structural models of K(+) channels. *Proc Natl Acad Sci U S A*. 103:7292-7297.

21. Simons KT, Bonneau R, Ruczinski I, Baker D (1999): Ab initio protein structure prediction of CASP III targets using ROSETTA. *Proteins*. Suppl 3:171-176.

22. Jo S, Kim T, Im W (2007): Automated builder and database of protein/membrane complexes for molecular dynamics simulations. *PLoS One*. 2:e880.

23. Case DA, Babin V, Berryman JT, Betz RM, Cai Q, Cerutti DS, *et al.* (2014): AMBER 14. University of California, San Francisco.

24. Dickson CJ, Madej BD, Skjevik AA, Betz RM, Teigen K, Gould IR, *et al.* (2014): Lipid14: The Amber Lipid Force Field. *J Chem Theory Comput.* 10:865-879.

25. Humphrey W, Dalke A, Schulten K (1996): VMD: visual molecular dynamics. *J Mol Graph.* 14:33-38, 27-38.

26. Gotz AW, Williamson MJ, Xu D, Poole D, Le Grand S, Walker RC (2012): Routine microsecond molecular dynamics simulations with AMBER on GPUs. 1. Generalized born. *J Chem Theory Comput.* 8:1542-1555.

27. Powell JA, Petherbridge L, Flucher BE (1996): Formation of triads without the dihydropyridine receptor alpha subunits in cell lines from dysgenic skeletal muscle. *J Cell Biol*. 134:375-387.
